# Re-thinking the possible interaction between proton pump inhibitors and capecitabine

**DOI:** 10.1007/s00280-022-04473-9

**Published:** 2022-09-13

**Authors:** Soo Hee Jeong, Lara Molloy, Edmond Ang, Nuala Helsby

**Affiliations:** 1 Molecular Medicine and Pathology, Faculty of Medical and Health Sciences, University of Auckland, Auckland, New Zealand; 2 Cancer and Blood Research, Auckland District Health Board, Auckland, New Zealand

**Keywords:** Capecitabine, Proton pump inhibitors, Drug–drug interaction, Treatment outcomes

## Abstract

Proton Pump Inhibitors (PPI) rank within the top ten most prescribed medications in Europe and USA. A high frequency of PPI use has been reported amongst patients undergoing chemotherapy, to mitigate treatment-induced gastritis or gastro-oesophageal reflux. Several recent, mostly retrospective, observational studies have reported inferior survival outcomes among patients on capecitabine who concomitantly use PPI. Whilst this association is yet to be definitively established, given the prominence of capecitabine as an anti-cancer treatment with multiple indications, these reports have raised concern within the oncological community and drug regulatory bodies worldwide. Currently, the leading mechanism of interaction postulated in these reports has focussed on the pH altering effects of PPI and how this could diminish capecitabine absorption, leading to a decrease in its bioavailability. In this discourse, we endeavour to summarise plausible pharmacokinetic interactions between PPI and capecitabine. We provide a basis for our argument against the currently proposed mechanism of interaction. We also highlight the long-term effects of PPI on health outcomes, and how PPI use itself could lead to poorer outcomes, independent of capecitabine.

## Background

Capecitabine, an oral prodrug of 5-fluorouracil (5-FU), is widely used in the management of gastrointestinal and breast cancer. Patients on capecitabine may concomitantly receive proton pump inhibitors (PPI) for the treatment of pre-existent or chemotherapy induced gastritis/gastro-oesophageal reflux disease. It is estimated that between 20 and 55% of patients with cancer use PPI [1]. A pharmacological interaction between capecitabine and PPI has been proposed, but available evidence remains inconsistent and confined to retrospective observational studies. Despite this, two drug information databases, Lexicomp and Micromedex, have updated their information on concurrent capecitabine and PPI use to include this possible interaction.

## Summary of current evidence

Several publications have raised concerns about the potential drug interaction between capecitabine and PPI leading to poorer patient outcomes. A recent systematic review concluded that there was conflicting evidence across the nine studies available at that time [2].

### Worse outcome

The first two studies that raised concerns about the possibility of a drug-drug interaction leading to inferior survival outcomes were a retrospective study of adjuvant capecitabine monotherapy in colorectal cancer patients [3] and the post-hoc analysis of the TRIO-013 study [4]. There was a lower five-year recurrence-free survival (RFS) rate among 298 colorectal cancer patients on adjuvant capecitabine monotherapy who were using PPI (74% vs 83%, *p* = 0.03) [3]. An inferior median progression free survival (PFS; 4.2 vs 5.7 months, *p* < 0.001) and overall survival (OS; 9.2 vs 11.3 months, *p* = 0.04) was also observed among 545 trial participants who were on concomitant PPI in the capecitabine and oxaliplatin (CapeOx) arm of the TRIO-013 trial [4].

Following this, several other reports have emerged. A retrospective study of 70 capecitabine treated patients indicated that concomitant acid suppression therapy decreased PFS (HR 2.24, 95% CI 1.06–4.41, *p* = 0.035) when adjusted for disease severity and age [5]. Furthermore, a retrospective review of 389 patients with stage II–III colorectal cancer showed a significant negative effect on three-year RFS inpatients using concomitant PPI when compared to non-users when treated with capecitabine-oxaliplatin CapeOx (69.5 vs 82.6%, *p* = 0.029). These patients also had an increased cancer recurrence or death (HR 2.03, 95% CI, 1.06–3.88; *p* = 0.033). In contrast, the 5-FU based (FOLFOX) treated PPI recipients had a trend to increased 3-year RFS compared to non-PPI users (82.9 vs 61.7%, *p* = 0.066) and decreased recurrence/death (HR 0.51, 95% CI, 0.25–1.06; *p* = 0.071) [6].

A recent analysis of 606 stage II–III colorectal cancer patients receiving capecitabine-based adjuvant chemotherapy found that 8.9% of patients received co-administration of PPI [7]. This was associated with a poor RFS and OS, although not statistically significant. However, using a propensity score-adjusted analysis using a logistic regression model, they found that patients treated with PPI had significantly shorter RFS (increased risk: 37–54%) and OS (increased risk: 12–26%) relative to patients treated without PPI.

### Possible improved outcome (PPI-5FU interaction)

In addition, to the non-significant trend to improved three-year RFS in patients treated with the FOLFOX regimen noted above [6] a post-hoc analysis of 482 patients in the Asian XELIRI ProjecT (AXEPT) randomised phase III trial showed that among PPI users, capecitabine containing treatment (mXELIRI: capecitabine and irinotecan) had poorer OS compared to the infusional 5-FU group (FOLFIRI: leucovorin, fluorouracil, and irinotecan), 16 vs 19 months, *p* = 0.15 [8]. However, in non-PPI users, the OS was better in the mXELIRI arm compared to the FOLFIRI arm (16 vs 15 months, *p* = 0.044) [8]. Importantly, when patients receiving mXELIRI were stratified according to PPI use, there was a non-significant trend towards better outcomes for patients receiving FOLFIRI with PPI.

Furthermore, a retrospective analysis [9] of 5-year outcomes in *n* = 671 stage IV colorectal cancer patients receiving CapeOX found no significant effect of PPI use on PFS and OS. However, significantly improved PFS (RR 0.67, 95% CI 1.10–2.05, *p* = 0.01) and OS (RR 0.72, 95% CI 1.02–1.90, *p* = 0.04) was observed in the PPI users receiving FOLFOX regimen. It is of note that usage of PPI was very high in this study with 84.4% of the 307 patients in the FOLFOX group receiving concomitant PPI and 59.1% of the 364 patients in the CapeOX group PPI users.

### Possible worse outcome (PPI-5FU interaction)

More recently, by accessing data from six clinical trials through data-sharing platforms, a secondary analysis of the effect of concomitant PPI use on fluoropyrimidine-based chemotherapy outcomes was undertaken [10]. Data from eleven arms across six randomised controlled trials involving > 5000 patients with advanced colorectal cancer was available. The proportion of PPI users in these trials ranged from 11.3–25.8%. Pooled analysis showed that PPI use was significantly associated with worse PFS (adjusted HR 1.20, 95% CI 1.05–1.37; *p* = 0.009) and OS (adjusted HR, 1.20, 95% CI 1.03–1.40, *p* = 0.02). Of note acid suppression due to receipt of histamine H2-receptor antagonists (6.8% of the participants in these six trials) had no effect on OS, PFS and response rates. This suggests a likely pharmacodynamic interaction rather than a pH-based alteration in pharmacokinetics (see later section). However, only one of the six trials in this post-hoc analysis was in patients receiving capecitabine and hence this data suggests that there may also be a previously under-appreciated interaction between 5-FU and PPI. Indeed, comparison of the effect of PPI use in the 980 patients receiving capecitabine versus the 4,282 receiving infusional 5-FU based schedules found no effect on OS in the capecitabine group (adjusted HR 0.92, 95% CI 0.75–1.12, *p* = 0.40). This contrasted with the significant effect of PPI use in the 5-FU group (adjusted HR 1.20, 95% CI 1.03–1.40, *p* = 0.02).

In a study of 508 locally advanced rectal cancer patients receiving either capecitabine or 5-FU based neoadjuvant chemoradiation (NACRT) [11], concomitant PPI use was 9.8% and 10.2 in both groups respectively. There was no significant effect of PPI use on disease-free survival and OS for capecitabine-based NACRT. However, in the patients receiving 5-FU there was a significant negative effect of PPI use on local recurrence (*p* < 0.005) and OS at both 36 and 60 months (*p* = 0.007). This significant effect on local recurrence was also observed in those PPI users who received 5-FU adjuvant therapy after surgery.

### Improved outcome (PPI-capecitabine)

In a study of 125 patients with rectal cancer [12] of which 50% had received defined doses of omeprazole at the time of neoadjuvant treatment, PPI users were reported to have improved response (OR 2.34, 95% CI 1.12–4.85, *p* = 0.02) to neoadjuvant RT-CapeOX. This study also postulated that there may be a dose cutpoint (total dose 200 mg) that differentiated disease recurrence. Using this cutpoint, response in PPI users was further analysed. Patients who received < 200 mg total dose omeprazole had worse disease-free survival (HR 0.30, 95% CI 0.90–0.97, *p* = 0.044). This data suggests that PPI use improves capecitabine outcomes in this therapeutic context and that there is a concentration dependent effect of PPI.

### No association of PPI use and outcome

In a study of locally advanced rectal cancer (*n* = 149, 15% of whom were on PPI) receiving capecitabine-based neoadjuvant chemoradiotherapy (NARCT) the complete pathological response rate was lower (8.7% versus 19%) in the PPI group. However, this was not statistically significant and there was also no significant difference in the recurrence-free rate or overall survival in the patients who used PPI during treatment [13]. Importantly, this study focussed on neoadjuvant capecitabine chemoradiotherapy and did not consider assessment of the effect of PPI on the subgroups of patients who then received either 5-FU-based (*n* = 53) versus or capecitabine based (*n* = 34) chemotherapy.

A number of other studies have also found no association between patient outcomes and PPI use in patients treated with capecitabine. This includes two reports, which were investigating the possible therapeutic benefit of high dose PPI as combination therapy (a phase II trial and a post-hoc analysis of a phase III trial), that have only been disseminated in abstract form [14, 15]. Another study of 72 Chinese patients, only available as an abstract in English [16], reported no effect of PPI use on objective response rate and PFS to capecitabine.

The inconsistency between studies described above, was highlighted in a systematic review [2], which included nine of the fourteen studies listed above, but did not include more recent publications [7, 8, 10, 11, 13]. We have briefly summarised the outcomes reported in these twelve studies for either capecitabine or 5-FU (Table 1). It is apparent that the likelihood of a drug-drug interaction between capecitabine and PPI remains unclear and that an interaction with 5-FU is also possible and has not previously been highlighted.

**Table 1:** Summary of the heterogeneity of the reported effects of concomitant proton pump inhibitor (PPI) use in patients receiving capecitabine or 5-fluorouracil (5-FU) based chemotherapy

References	Study size (*n*)	Capecitabine + PPI versus no PPI	5-FU + PPI versus no PPI
Sun et al. (2016) [3]	298	Worse outcome	NA
Chu et al. (2017) [4]	545	Worse outcome	NA
Rhinehart et al. (2019) [5]	70	Worse outcome	NA
Kitazume et al. (2022) [7]	606	Worse outcome	NA
Wong et al. (2019) [6]	389	Worse outcome	Non-significant trend to improved outcome
Kim et al. (2021) [8]	482	No effect^a^	Non-significant trend to improved outcome
Wang et al. (2017) [9]	671	No effect	Improved outcome
Kichenadasse et al. (2021) [10]	5,262	No effect	Worse outcome
Menon et al. (2021) [11]	508	No effect	Worse outcome
Zhang et al. (2017) [12]	125	Improved outcome	NA
Bridoux et al. (2022) [13]	149	No effect	Not addressed
Roberto et al. (2019) [14]	61	No effect	NA
Yang et al. (2017) [15]	891	No effect	NA
Lu et al. (2019) [16]	72	No effect	NA

*NA* not applicable

^a^This study reported significantly worse outcomes for PPI users receiving capecitabine compared to those receiving 5-FU plus PPI

It is also of interest that chemotherapy associated-adverse events may be lower when PPI are combined with capecitabine. An experiment in mice showed that the rate of hand-foot syndrome, a dose-limiting toxicity of capecitabine, was lower in the group that were given capecitabine with omeprazole (6.25% vs 8.31%, *p* < 0.0001), [17]. The same article analysed the clinical database from the US Food and Drug Administration (FDA) Adverse Events Reporting System and found that the rate of capecitabine-related hand-foot syndrome was lower in the group that used PPI than in the group that did not (6.25% vs 8.31%, *p* < 0.0001). PPI may have anti-inflammatory effects [18] and this could influence the extent of this adverse event. However, this toxicity is related to the pharmacological activity of capecitabine and may offer indirect evidence of the ability of PPI to decrease the activation of capecitabine.

Few clinical studies report the specific PPI used or confirm the duration of use during chemotherapy. Hence it is not known if any particular PPI has more or less propensity to cause drug-drug interaction.

Although multiple mechanisms of interaction have been proposed, PPI-mediated gastric pH changes leading to decreased absorption of capecitabine has been the most popular [4]. Previous studies have shown the absorption and bioavailability of capecitabine can be variable, and its pharmacokinetics can be affected by food [19]. Since capecitabine is orally administered, gastric pH may potentially alter the dissolution and absorption of capecitabine. It is therefore worthwhile to explore whether this is the likely explanation for the potential interaction between capecitabine and PPI, if any.

## Impact of PPI on capecitabine dissolution

Capecitabine is believed to have good permeability and is readily absorbed in the small intestine as soon as it has dissolved [19]. The nature of the solvent used to dissolve a drug can significantly alter the rate of dissolution. Altering the pH of the stomach through PPI use, would likely impact this rate. This is the mechanism multiple authors have proposed to explain the decreased RFS or PFS retrospectively observed following concurrent PPI and capecitabine use [3, 4, 20]. This also appears to be supported by data from in vitro studies by Hoffmann-La Roche, Ltd, showing that capecitabine tablets can take longer to dissolve in a basic solution [19]. However, this data is not readily available to validate. Moreover, PPI use is not expected to increase gastric pH to the extent that a basic environment is produced within the stomach, instead use of PPI simply results in a less acidic pH. Reigner et al. [19] only comment on the low stability (i.e. degradation) of capecitabine at low pH rather than the effect of increasing pH on the extent of dissolution. Alternative data from capecitabine dissolution studies in vitro show that over 85% will dissolve within 30 min in the pH range of 2.0–6.8 [21]. Since the highest gastric pH likely to be reached with PPI use is pH 5 [20], this suggests PPI use would not alter capecitabine dissolution enough to significantly change exposure (plasma AUC) to the drug. Moreover, a study investigating the pharmacokinetics of capecitabine as an oral suspension, compared to tablet form, found that although there was a higher maximum concentration (Cmax) for capecitabine and 5-FU when given as an oral suspension, the AUC of both compounds were bioequivalent following oral suspension and tablet formulation [22]. This would suggest that whilst the rate of dissolution may impact the Cmax of capecitabine, it is unlikely to significantly change overall plasma AUC exposure, and therefore effectiveness, of the drug.

## Impact of PPI on capecitabine absorption

Orally administered drugs must be absorbed from the gastrointestinal (GI) tract, either by passive diffusion across cell membranes or facilitated transport using a transport protein. For a drug to diffuse across a cell membrane it must be unionised. This will depend on pH, i.e. an acidic drug will be unionised in an acidic environment and a basic drug will be unionised in a basic environment. Although acidic drugs will be unionised in the stomach, passive absorption in the stomach is considered low compared to the small intestine, where the majority of drug absorption will occur due to the large surface area [23]. The pH in the small intestine increases distally, thus acidic drugs will be absorbed across the proximal, rather than distal intestine, with a shorter time to maximal plasma concentrations (Tmax) than basic drugs [24]. Capecitabine is a weak acid with a pKa of 8.8, meaning it is unionised at low pH [20]. It has a partition coefficient of 0.4, suggesting it is moderately lipophilic and will therefore be readily absorbed when unionised in an acidic environment [19, 25]. Using the Henderson-Hasselbach equation and the pKa of capecitabine, it is possible to calculate the percentage of drug ionised at various pH values. Based on a stomach pH of 5, the maximum increase induced by PPI [20], at this pH the amount of ionised capecitabine is negligible (less than 0.1%). This suggests that decreased capecitabine absorption pharmacokinetics due to changes in ionisation with concomitant PPI use are unlikely.

The absorbance of capecitabine in patients who have undergone either a partial or total gastrectomy is also of interest in this context. A study has found that gastrectomy patients have an increased rate of capecitabine absorption [26], which may be explained by capecitabine tablets entering the small intestine at a faster rate. The AUC data was not reported in this study. However, it was found that patients who had received a gastrectomy did not require a dose adjustment, suggesting overall exposure was not substantially altered. This is of interest in relation to the previously proposed role of PPI-mediated pH changes in capecitabine absorption as a cause of negatively impact on patient outcomes. The average stomach pH in patients following a partial gastrectomy is much higher than the typical acidic pH in healthy patients, at approximately 7.0 [27]. Since capecitabine treatment outcomes were not significantly altered in partial gastrectomy patients, when gastric pH is expected to drastically increase, it would suggest this is not a mechanism by which PPI mediate decreased treatment outcomes.Gastric emptying rate can also alter the rate of drug absorption. A faster emptying rate will expose the drug to the small intestine sooner than a slower emptying rate so that the drug is likely to be absorbed faster [23]. Indeed, this explains why patients who have had a gastrectomy had an increased rate of absorption of capecitabine. Moreover solid food can decrease rate of gastric emptying, whereas liquids can increase it [28]. A study investigating a possible interaction between capecitabine and the antacid Maalox found that the Tmax of capecitabine absorption was lower when taken with 20 mL Maalox [29]. The study concluded there was unlikely to be an interaction between capecitabine and the antacid, as the AUC were not significantly different. Instead, a possible explanation for the decreased Tmax is that the liquid may have increased the gastric emptying rate, leading to capecitabine entering the small intestine faster, increasing the rate of absorption.

Assessment of taking capecitabine with food [19], found that patients in a fasted state had a higher capecitabine Cmax and AUC compared to those who consumed food with their dose. The AUC_0–∞_ ratio (Fasted:Fed) was 1.51 (90% CI 1.28–1.79) and it is not clear from this publication if this was a statistically significant change. However, this suggests that the decreased gastric emptying rate due to consumption of solid food decreased the rate of capecitabine absorption (Cmax and Tmax) and led to lower exposure (AUC). There is evidence that PPI can slow the gastric emptying of solids (Sanaka et al. 2010). Since it is advised that capecitabine is to be taken with food, concurrent PPI use could delay the rate at which the food is emptied, therefore delaying capecitabine reaching the small intestine, leading to slower capecitabine absorption and decreased AUC. Although there are no data regarding clinical outcomes of patients taking capecitabine in a fasted state, the similarity between AUC in fed or fasted states for the key active metabolite, 5-FU [19], suggests patient outcomes would not be affected.

## Impact of PPI on capecitabine pharmacokinetics

There is a paucity of data on the effect of PPI on the pharmacokinetics of capecitabine, until recently there was only one published study that had attempted to investigate this interaction. This study investigated the plasma concentrations of capecitabine and its metabolites in patients taking the PPI rabeprazole compared to a separate control group [30]. No significant difference in pharmacokinetics was found. However, this was limited by the large inter-individual variation of plasma profiles with each of the two groups, as well as unequal sample size (five patients in the PPI group and nine in the control group).

Recently a well-designed randomised cross-over trial of capecitabine and esomeprazole in 22 patients has been reported [31]. Whilst there was an increase in the geometric mean AUC and Cmax of capecitabine by 18.9% and 9.9%, respectively after esomeprazole administration, these changes were not statistically significant. The consumption of an acidic cola beverage also had no significant effect on the rate or extent of capecitabine absorption pharmacokinetics. However concomitant PPI significantly increased the median half-life of capecitabine (0.63 versus 0.46 h, *p* = 0.005). This suggests that PPI decrease the elimination clearance of capecitabine. The authors did not determine the pharmacokinetics of the formation of the sequential metabolites of capecitabine (5’-deoxy-5-fluorocytidine and 5’-deoxy-5-fluorouridine) but did assess the active metabolite 5-FU. Plasma concentrations (AUC_0-inf_) of 5-FU were 7.8% higher after esomeprazole compared to no PPI (406.7 vs 385.9 ng.h/mL), although this was not statistically significant. The plasma half-life of 5-FU was also increased after esomeprazole (0.88 h versus 0.76 h), i.e. a similar effect on elimination to that observed for capecitabine. This pharmacokinetic data refutes the theory that PPI decrease capecitabine absorption. However, the ability of esomeprazole to apparently delay the plasma elimination of capecitabine as well as 5-FU requires further investigation. To gain a clearer picture the impact of PPI on not only capecitabine pharmacokinetics, but also the formation of the of sequential intermediate metabolites as well as the active metabolite 5-FU and also the effect on renal elimination processes, more randomised cross-over trials are required.

## PPI use and health outcomes

### Effects on the kidney

PPI use may be associated to adverse effects on the kidney, since PPI are known to be one of the most common causes of drug-induced acute interstitial nephritis [32]. The exact mechanism by which PPI use leads to acute interstitial nephritis is currently unknown. PPI, such as omeprazole, are eliminated from the body via hepatic clearance catalysed by the genetically polymorphic CYP2C19 enzyme. However, there is no relationship between inherited loss of function CYP2C19 and this type of kidney damage [33]. More importantly, a large prospective cohort study of more than 10,000 adults found that PPI use was associated with a 20–50% higher risk of chronic kidney disease [34]. It is of note that for patients with moderate renal impairment (30–50 mL/min), capecitabine dosage adjustment is recommended, and its use is contraindicated in patients with poor renal function (< 30 mL/min, [35] due to increased risk of toxicity. Renal excretion is important in the elimination of the active metabolite 5-FU (particularly if hepatic clearance by dihydropyrimidine dehydrogenase is impaired). If PPI can cause sub-clinical damage to the kidney, the concomitant use of PPI with capecitabine could alter the renal elimination of capecitabine (and its active metabolites) and alter therapeutic outcomes.

### Effects on the gut microbiota

Long term PPI use may lead to alterations in gut microbiota, which can in turn increase the susceptibility of various GI disorders [36]. Because gut microbiota is involved in many physiological functions (e.g. homeostasis, metabolism, inflammation and immunity), disruptions to gut microbiota may also impact cancer therapy response and risk of adverse effects [37].

### All-cause mortality

It is of note that there is increasing concern that PPI use is associated with increased risk of all-cause mortality and also an increased risk of cancer diagnosis. A large cohort study (including 689,602 PPI users) found all-cause mortality was higher in patients prescribed PPI (weighted HR 1.38, 95% CI 1.33–1.44), [38]. If this is a real association, then the potentially worse outcomes in patients treated with capecitabine concomitant with PPI may be explained by the increased risk of mortality due to the PPI alone, rather than a pharmacological interaction between the two drugs. Another large cohort study (*n* = 32,411) found that PPI use after colorectal cancer diagnosis was associated not only with increased all-cause mortality (adjusted HR 1.38, 95% CI 1.32–1.44) but also colorectal cancer-specific mortality (adjusted HR 1.34, 95% CI 1.28–1.41), [39]. Furthermore, long-term use of PPI may also influence the risk of cancer development. A Swedish cohort study (including 738,881 PPI users) reported that standardised incidence ratios for gall bladder, extrahepatic and intrahepatic bile duct cancer were 1.58 (95% CI, 1.37–1.81), 1.77 (95% CI, 1.56–2.00), and 1.88 (95% CI, 1.57–2.23) respectively, when long-term (≥ 180 days) PPI users were compared to the general population [40]. A population-based prospective cohort study in South Korea reported that, in a low risk population (non-obese, non-diabetic females under 50 years of age, no history of alcohol consumption), the risk of colorectal cancer was higher in PPI users than non-users (adjusted HR 12.30, 95% CI 1.71–88.23), [41]. PPI use was also found to be associated with a higher risk of colorectal cancer in a Taiwanese cohort study that included 45,382 PPI users (adjusted HR 2.03, 95% CI 1.56–2.63, *p* < 0.001), [42]. However, these studies should be interpreted with caution since PPI may be given to people with other underlying health conditions who may already have increased risk of death or cancer development, particularly as PPI use may also be more common in older patients [38]. Indeed a recent systematic review has highlighted that epidemiological studies do not support an increased risk of developing colorectal carcinoma in PPI users [43]. That systematic review also highlights that basic science and preclinical research suggests that paradoxically PPI have anti-tumour properties.

## Conclusion

The question of PPI-capecitabine interaction and its potential effects on anti-cancer efficacy, and consequently survival, is an important one with implications on practical, day-to-day cancer care worldwide. If an efficacy reducing interaction truly exists, patients needing PPI may be better served using an alternative anti-cancer treatment to achieve best treatment outcomes.

It is important to consider that the premise of this interaction is currently limited to largely retrospective studies and post-hoc analysis of trials and is by no means definitive. Importantly, there is no evidence to suggest that PPI will adversely affect the dissolution or absorption of capecitabine. In this article, we have undertaken the task of revisiting the question of PPI-capecitabine interaction with a wider lens. We suggest taking this high impact and clinically relevant question from the bedside, back to the bench for further scrutiny. In addition, we believe the scope of investigation should be broadened to study other alternative drug-drug interaction mechanisms, such as altered renal elimination or potential effects on transport of capecitabine’s active metabolites into tumour cells. This should be investigated in well-designed pharmacokinetic and therapeutic outcome studies. Lastly, the effects of PPI on long-term health outcomes require further elucidation.

## Data Availability

Not applicable.
